# Temperature‐Assisted Crystallization of Mustard Oil for Erucic Acid Reduction and Its Impact on Phenolic Compounds, Sterols and Oxidative Quality of Oil During Ambient Storage

**DOI:** 10.1002/fsn3.71883

**Published:** 2026-05-18

**Authors:** Muhammad Abdul Rahim, Muhammad Nadeem, Mohamed Fawzy Ramadan, Eliasse Zongo, Layla A. Alahmari

**Affiliations:** ^1^ Department of Food Science & Nutrition Times University Multan Punjab Pakistan; ^2^ Department of Dairy Technology University of Veterinary and Animal Sciences Lahore Punjab Pakistan; ^3^ Department of Clinical Nutrition, Faculty of Applied Medical Sciences Umm Al‐Qura University Makkah Saudi Arabia; ^4^ Laboratory of Research and Teaching in Animal Health and Biotechnology Universite Nazi Boni Bobo‐Dioulasso Hauts‐Bassins Region Burkina Faso; ^5^ Department of Community Health, College of Applied Medical Sciences Northern Border University Arar Saudi Arabia

**Keywords:** erucic acid, mustard oil, oxidative stability, phenolic compounds, temperature induced crystallization

## Abstract

Mustard oil (MO) is produced from non‐genetically modified seeds of mustard, a member of the *Brassicaceae* family. MO contains about 40% erucic acid (EA), which is perceived to have negative health impacts. To make MO a health friendly oil, EA was first time reduced by temperature‐assisted crystallization. MO was slowly cooled to −28°C (12 h) and held at this temperature for 16 h. Solid and liquid phases were separated from each other by vacuum filtration (−600 mmHg pressure, Buchner Funnel). Filtrate was named as olein (OP), residue (over filter paper) was designated as stearin (SP), followed by packaging in brown glass bottles and storage at 25°C–30°C for the duration of 3 months. Gas chromatographic analysis showed that EA (C22:1) in OP was 1.97 mg/100 g (*p* < 0.05). While, the contents of EA in MO and SP were 41.23 and 78.91 mg/100 g, respectively. HPLC characterization revealed that temperature‐assisted crystallization had a great impact on the distribution of phenolic compounds from MO to OP and SP. OP had the highest concentration of phenolic compounds, followed by MO and SP (*p* < 0.05). In OP, sinapic acid, *p*‐coumaric acid, ellagic acid, and cinnamic acid were 132.57, 236.19, 188.72, and 95.62 mg/100 g. At the end of the storage phase of 3 months, the free fatty acids (FFA) contents of OP and SP were 0.17% and 0.18%, respectively (*p* > 0.05). Temperature‐assisted crystallization did not affect the sensory quality, color, and smell characteristics of MO, which were retained in OP and SP (*p* > 0.05). Temperature‐assisted crystallization can be used to lower EA in MO with reasonable oxidative stability and acceptable sensory characteristics.

## Graphical abstract

Erucic acid (EA) content of mustard oil (MO) was decreased to < 2% level using temperature‐assisted crystallization with no effect on free fatty acids (FFAs), peroxide value (PV), and color of MO and its olein and stearin variant. Olein was rich in phenolic compounds and had higher antioxidant capacity than MO and stearin. After the completion of storage phase, FFA and PV of OP (low EA variant) were within the limits of European Union.

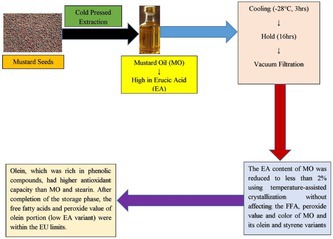

## 1 Introduction

Over the last few decades, oilseed crops have attracted more attention from researchers due to their significant role in human nutrition, economic importance, and global food security perspectives. Several genotypes of oilseed crops were developed/modified to increase yield or chemical characteristics to feed the ever‐increasing human population (Suryawanshi et al. 2025). World Health Organization estimates suggest an increase of more than two billion people in Asian and African continents (World Health Organization 2008). Several hundred edible oils have been discovered by scientists however, edible oil industries are processing only five to six edible oils on a larger scale (Hamm et al. 2013). Reason for non‐processing of edible oils and applications in commercial food products may be due to availability, cultural or social issues, and chemical composition of the oil or the presence of chemical compounds which are perceived to have adverse health effects (Galanakis 2020). Mustard oil (MO) is a member of the *Brassicaceae* family, it is one of the most popular edible oils in the worlds, consumption wise, it holds third position after palm oil and soybean oil in the world with an average production of 10.2 million tons annually (Chauhan et al. 2011). Concentrations of unsaturated fatty acids (USFAs) and bioactive compounds are considerably higher in MO than in fats of animal origin. It is one of the most commonly used edible oils in India and China, with an estimated utilization of 25–30 million tons annually (Ullah, Nadeem, Ayaz, et al. 2016). Adaptability in a diversified climate, higher oil content, and application in several traditional foods make MO a critical determinant of the oil market. Chemical characteristics of edible oils, such as fatty acids profile, triglycerides composition, tocopherols, and erucic acid (EA) contents, greatly impact the quality traits of MO. In MO, average concentrations of C16:0, C18:0, C18:1, C18:2, C18:3 and C22:1 were 1.2, 1.6, 45.7, 8.3, 40.9%, respectively (Athanasiadis et al. 2024). EA (C22:1) a monounsaturated fatty acid, double bond (*cis* form) is positioned at the 9th carbon atom from the methyl end. EA intake has been connected to myocardial lipidosis and heart lesions; oils having high EA content are perceived as unhealthy for humans (Nadeem, Imran, Iqbal, et al. 2017). Oil processing industries of the world are producing several versions of cold pressed and refined, bleached, and deodorized cooking oils (Gharby 2022; Rahim et al. 2023). Partially hydrogenated fats for the production of bakery shortenings from almost all commercially available oils however, MO still has not become a part of industrially processed oils despite being a huge source of edible oil due to the high content of potentially harmful EA (about 40%). EA has been declared a natural toxicant, and its limits have been regulated in many countries. European Union and Joint Food Standard Code of Australia and New Zealand allow 5% and 2% EA, respectively (FDA 2016; Vetter et al. 2020). Canola, a low EA version of rapeseed oil has been developed, most of the high EA rapeseed oil is consumed in developing countries (Kramer 2012). The science of genetics in developing countries is at primitive stages and not developed to the level where genetic modification of oil producing crops can be done at the gene level, Further, no industrial method has been devised by the researchers to reduce the EA to safe limits. Replacement of high erucic rapeseed varieties with low EA varieties on millions of hectares of land using imported low EA rapeseed varieties is practically impossible and unsustainable. Therefore, despite of the invention of low EA variants, high EA varieties are extensively grown and MO with higher EA content is consumed by the billions of human beings. In addition to the genetic modification, technological method for decreasing EA from high EA rapeseed oil should be discovered. Technologically, fatty acid profile of edible oils can be modified by *transesterification*, interesterification, blending, fractionation also known as winterization/dry crystallization etc. (Azeem et al. 2015). Technically, fractionation is based upon the difference in melting points of fatty acids, monounsaturated, polyunsaturated and saturated fatty acids have different melting points and crystallize at different temperatures. Melting point of EA varies from 28°C to 32°C while melting point of oleic acid and linoleic acid are 4.4°C and −5°C, respectively. This tremendous variation in melting point among the fatty acids of EA may be capitalized to technologically decrease/remove EA from MO. In a recent study, safflower oil was fractionated at −45°C, and concentrations of USFAs in safflower oil, mid and olein fractions were 62.4%, 3.8% and 87.9%, respectively (Khan et al. 2024). Fractionation of MO to produce olein and stearin has not been previously reported. Therefore, a comprehensive study needs to be conducted to study the anticipated chemical changes. As a low EA fraction will be developed for applications in food industries, its oxidative stability also needs to be analyzed. At industrial scale, solvent and controlled temperature crystallization techniques are used to produce different fractions of oils for diversified applications, from safety viewpoints, the controlled temperature crystallization technique is safe as it does not involve the usage of solvents (Alim et al. 2008). Naturally existing bioactive compounds and tocopherols are lost during high temperature deodorization (230°C–240°C) for 3–4 h further, this harsh processing, generates some undesirable trans fatty acids and polymers. Therefore, nutritionist recommend to use cold pressed oils or avoid harshly processed edible oils. Edible oil processing involves enormous amount of energy, for converting 1 metric ton of crude oil to a refined, bleached and deodorized (RBD) product, 2 metric tons of saturated steam are required. By reducing EA content in MO, it may be used in virgin and extra‐virgin forms however, a detailed investigation should be performed to develop a sustainable and environment friendly method for the development of low EA MO.

## 2 Materials and Methods

### 2.1 Experimental Plan

*Brassica juncea* seeds were mechanically pressed by screw press at 25°C–30°C, cold expressed (21.4% of oil content) was stored in clean and dry amber glass bottles and used in this experiment within 3 days of production. MO was gradually cooled to −28°C (3 h) and then it was held at this temperature for 16 h. Solid and liquid phases were separated from each other by vacuum assisted filtration assembly (Buchner Funnel). Filtrate was named as olein portion (OP), residue (over filter paper) was designated as stearin portion (SP). The process of fractionation was repeated six times using the same oil source, equipment, temperature conditions, vacuum pressure (−600 mmHg) using vacuum pump, followed by packaging in brown glass bottles and storage at 25°C–30°C for the duration of 3 months. All parameters were analyzed at 0, 45, and 90 days.

### 2.2 Chemical Properties MO, OP, and SP

Free fatty acids (FFA), moisture content (MC), iodine number (IN), saponification value (SV), unsaponifiable matter (UM), and peroxide value (PV) were determined (Association of Official Analytical Chemists 2000). Color was checked on Lovibond Tintometer in 5.25‐inch quartz cell.

### 2.3 Fatty Acid Profile (FAP)

Oil sample 50 mg was treated with a mixture (98 mL C_2_H_5_OH and 2 mL H_2_SO_4_) at 100°C/60 min in test tubes, followed by cooling, addition of each 2 mL *n*‐hexane and double distilled H_2_O, 20 min resting and extraction to GC‐vials and 1 μL was injected by ALS into column‐SP2560 (100 m × 0.25 mm ID, 0.20 μm film thickness) using GC–MS (7890‐B). Temperature ramping was as follows: 10°C/min to 150°C, holding time 12 min, 15°C/min to 250°C with 13 min holding time, maintaining injector and FID at 250°C in a steady stream of He, H_2_ and O_2_ (2.5, 50 and 5 mL each) using FAME‐37 standard (Qian 2003).

### 2.4 Phenolic Compounds of MO

Sample oils (10 g) were dissolved in 10 mL *n*‐hexane, the upper layer was extracted three times with 20 mL C_2_H_5_OH (60%), followed by removal of excess solvent on a rotary evaporator, and 20 μL was injected into HPLC (Shimadzu) fitted with LC‐8A chromatography pump, DAD, SPD‐M10A VP detector, reverse phase column. C_2_H_5_OH, CH_3_COOH, and H_2_O (90:2:8, respectively) constituted the mobile phase at the following rate of 1 mL/min at 280 nm with using six standards of each phenolic compounds; sinapic acid and cinnamic p‐coumaric acid, ellagic acid and cinnamic acid from MO on a calibration curve (Thiyam‐Holländer et al. 2014).

### 2.5 Phytosterols in MO

Brassicasterol, Campesterol, Campestanol, β‐Sitosterol, Sitostanol, Δ^5^‐Avenasterol, Cycloartenol, and 24‐Methylene cycloartenol were determined. Briefly, an oil sample (0.3 g) was reacted with 10 μL (5α‐Cholestane 0.5 mg/mL as an internal standard) and ethanolic NaOH (2 M, 3 mL) at 90°C for 15 min in a water bath; each 2 mL of distilled H_2_O and *n*‐hexane were added to the test tube and centrifuged at 5000 *g* for 15 min. Supernatant was reacted with Tri‐Sil (10 μL) for 30 min under N_2_ and shifted to injection vials. Sample, 1 μL was injected into column (30 m × 0.32 mm × 0.1 μm), temperatures of injector and FID were 265°C, oven temperature was increased 5°C/min till 300°C, in the presence of He, H_2_ and O_2_ (2.5, 50 and 5 mL each) using internal standards (Azadmard‐Damirchi 2010).

### 2.6 Total Antioxidant Capacity (TAC)

Each Na_2_SO_4_ (28 mM), (NH_4_)_2_MoO_4_ (4 mM), and H_2_SO_4_ (6 M) was mixed and reacted with 100 μL sample at 95°C for 15 min, followed by cooling and determination on a double‐beam spectrophotometer at 695 nm; TAC was described in percentage (Wolfe et al. 2003).

### 2.7 1,1‐Diphenyl‐2‐Picrylhydrazyl (DPPH)

DPPH (20 mg) was dissolved in 1000 mL C_2_H_5_OH, a sample (750 μL) was mixed with 1500 μL of DPPH reagent, followed by 30 min incubation in the dark and determination on a double‐beam spectrophotometer at 517 nm, and the results were revealed in percentage (Wolfe et al. 2003).

### 2.8 Lipid Oxidation and Oxidative Stability

FFA and PV were determined in fresh, 45 and 90‐days old samples; 2.5 g oils were weighed in eight reaction vessels of Professional Rancimat (892), a constant water free oxygen was introduced from the bottom (20 L/h) at 120°C till the straight‐line breaks using Stab Net Software for the operation and auto calculation of induction period (AOCS 2011).

### 2.9 Sensory Evaluation

Samples of MO, OP, and SP were randomly coded and arranged; color and smell in all samples were evaluated using 10 trained judges (age ranges from 30 to 43 years) having previous experience in sensory evaluation of edible oils and fats using a 9‐point scale. Several training sessions were conducted to standardize the vocabulary. Unsalted bread and distilled water were also provided in each sensory evaluation booth. Evaluations were performed in a specialized laboratory in booths, and data were recorded by FIZZ software (Larmond 1977).

### 2.10 Statistical Analysis

Two‐way ANOVA was applied to the analysis of collected data (in CRD six times replication of each fraction and thrice analysis of each sample) to determine the impacts of temperature‐induced crystallization and storage. For significant variations, DMR test was used to find the significant variations at a *p*‐value of 0.05 in SAS 9.4.

## 3 Results and Discussion

### 3.1 Chemical Characteristics of MO and Fractions

Alarming situation of food insecurity in Asian and African countries, diminishing sources of dietary lipids, prevalence of malnutrition has led the scientists to genetically or chemically modify the existing sources of oils and fats to improve the drastically deteriorating the situation of food security (Shahidi 2005). Crude MO is extensively used in the preparation of large number of culinary food items, pickling of fruits and vegetables etc. however, it could not become a member of industrially processed edible oils family mainly because of existence of > 40% EA, a fatty acid considered undesirable for the human cardiovascular system (FDA 2016; Singh 2018). With the advancement of lipid chemistry and development of several methods of fatty acid composition of oils and fats, fatty acid composition of MO can be modified and EA content can be reduced to the allowable limits and its industrially processed variants can be used in the production of partially hydrogenated feedstock's for the production of bakery shortenings, margarine and tailored fats. Among these methods, fractionation/dry crystallization/winterization is environmental, health friendly and sustainable method due to its simplicity, practicability and acceptance in edible oil processing sector the of the world (Gibon 2006). Oil content of most of the oilseeds is lesser than MO and these are industrially processed; MO has almost 50% more oil content than soybean. Consumption of unprocessed MO having EA content cannot be stopped with to its centuries old use in cooking and pickling etc. due to its typical flavor and aroma. Industrial processes such as refining, bleaching and deodorization remove all typical flavors from edible oils, refined, bleached and deodorized (RBD) MO cannot become an alternate of crude MO due to typical taste that has become the part of legacy of several traditional food items across the world. Therefore, in current study, only EA content of MO was decreased by temperature‐assisted crystallization with no refining, bleaching and deodorization, so that a low EA variant of MO can be developed for the consumers with no potential harmful effects of EA. This result has great deal of public and industrial interest as it has resolved a centuries old problem associated with the intake of high EA, MO. Moisture content of MO and its two created variants was less than 0.2%, and well within the permissible limits as far as crude oils are concerned, higher moisture content may cause undesirable hydrolysis of triglycerides leading to deterioration of market quality as price is based mainly on FFA content, excessive FFA lead to more NaOH, hot water, soap stock and ultimately higher refining losses (Hussain 2010). In this study, FFA content of MO was 0.14% Table 1 which is less than FFA content of soybean, sunflower and palm oil is reported in literature (Aladedunye and Przybylski 2014). In present investigation, impact of temperature‐assisted crystallization/fractionation did not show significant impact on FFA content of olein and stearin portions. In crude oils, FFA are either reduced by alkali or physical refining having no connection with lower temperatures employed in fractionation or dewaxing etc. Nadeem et al. (2015) performed fractionation of fat 10°C, 15°C and 25°C, to separate olein from the ice cream, no scientific correlation was found between fractionation and FFA content (*p* > 0.05). However, fractionation caused a pronounced variation in IN of OP and SP of MO. IN of MO, OP and SP were 146.82, 189.63 and 95.27 cg/100 g (*p* < 0.05). Iodine value of oleic acid, linoleic acid, linolenic acid and EA is 89, 181, 273 and 75 cg/100 g, variation in IN led to a major change in IN of OP and SP (O'Brien 2008). Phytosterols, tocopherols, pigments and phytochemicals constitute the UM in oils and fats, most of the UM constituting substances are lost in alkali refining of crude oils. Trend of intensification of UM in SP was observed, UM contents of MO, OP and SP were 1.35%, 1.67% and 0.95%, respectively. Low melting triglyceride have affiliation with unsaponifiable substances therefore, OP has significantly higher UM content. Earlier studies also reported the transfer of unsaponifiable substances from parent oil to the low melting glycerides during the fractionation (Shahidi 2005; Gomez‐Coca et al. 2015). Fractionation of chia oil was performed at −30°C for the enrichment of omega‐3, fractionation significantly altered the IN and UM content in olein and stearin of chia oil, with no effect on SV, color and FFA (Ullah, Nadeem, Khalique, et al. 2016). Color of oils is mainly due to chlorophylls and carotenes; these also affect the oxidation of oils (Mahoney et al. 2018). Cottonseed oil was fractionated to produce olein and stearin portions, major variations were recorded in IN, UM content of the hard and soft fraction (Azeem et al. 2015). Analytical examination of OP and SP showed that both met the requirement of the cold pressed standard of Codex Alimentarius (Hishamuddin et al. 2020).

**TABLE 1:** Chemical characteristics of MO, OP and SP.

Parameter	MO	OP	SP
Moisture content (%)	0.18 ± 0.02^A^	0.17 ± 0.01^A^	0.16 ± 0.08^A^
FFA (oleic acid, %)	0.14 ± 0.05^A^	0.13 ± 0.03^A^	0.15 ± 0.06^A^
IN (Wijs)	146.82 ± 2.36^B^	189.63 ± 1.48^A^	95.27 ± 1.94^C^
SV (mgKOH/g)	195.23 ± 0.18^A^	194.13 ± 0.62^A^	192.55 ± 0.44^A^
UM (%)	1.35 ± 0.09^B^	1.67 ± 0.04^A^	0.95 ± 0.12^C^
Color (Red+Yellow)	105 ± 1.58^A^	102 ± 0.89^A^	104 ± 0.08^A^

*Note:* Means of MO, OP and SP notified in a row with different letter, reveal statistically significant variation at 0.05 *p*‐value.

Abbreviations: MO, mustard oil; OP, olein; SP, stearin.

### 3.2 Fatty Acid Composition

Nutritional characteristics of dietary lipids is mainly based on their fatty acid profile; oxidative stability is also largely based on the presence of polyunsaturated fatty acids (PUFA). In this pioneer study, MO was subjected to temperature‐assisted crystallization at −28°C for the duration of 3 h to crystallize the EA for the production of low EA version for increasing its applications in foods and making it as an option for the edible oil producers to use it in the formulation of blended cooking oils and Vanaspati formulations. Temperature‐assisted crystallization induced major variations in fatty acid profile of OP and SP (*p* < 0.05). Like all other oils, MO is mainly composed of tri‐esters of glycerol and fatty acids having considerably different melting points (MP). On the basis of variation in MP of individual fatty acids, that is, C22:1, C18:3, C18:2, C18:1, C18:0, C16:0 and C14:0 was 33.4, −17.2°C, −12.4°C, 68.5°C, 63.5°C and 44.2°C (Nadeem et al. 2015). OP having < 2% EA was produced and saturated fatty acids were intensified in SP. Gas chromatographic analysis of MO showed the existence of C12:0 (0.49), C14:0 (2.63), C16:0 (4.49), C18:0 (3.27), C18:1 (21.84), C18:2 (13.95), C18:3 (8.11) and C22:1 (41.23) mg/100 g, respectively (Table 2). As a result of temperature‐assisted crystallization and subsequent filtration, high MP fatty acids such as C22:1 (EA), C18:0, C16:0, C14:0 and C12:0 were intensified in SP while, fatty acids with lower MP became the part of OP. Noticeably, extents of C22:1, C18:3, C18:2, C18:1, C18:0, C16:0 and C14:0 in OP were 1.97, 18.12, 30.76, 45.19, 1.29, 0.16, 0.09 and 0.13 mg/100 g (*p* < 0.05). OP had suitable amounts of C18:2 and C18:3 with lower contents of saturated fatty acids (< 2%). OP had 45.19% C18:1, which possesses cholesterol lowering with cardio‐protective effects (Rahman et al. 2014). Extents of C22:1, C18:3, C18:2, C18:1, C18:0, C16:0 and C14:0 in SP were 79.81, 0.23, 0.11, 0.44, 5.67, 6.97, 4.38 and 1.35 mg/100 g (*p* < 0.05). SP may be used for the production of laundry soap along with acid oils etc. It is for the first time that EA was reduced from MO by a technological method, for the food and oil industries, it is extremely useful method as winterization, fractionation, dewaxing or temperature‐assisted crystallization is already performed in almost all edible oil industries of the world. With lesser than 2% EA, MO can be included in the category of low‐erucic rapeseed oil. Further, this innovation will attract the edible oil and food processing industries to use low‐erucic MO to process in their refineries and food processing avenues. Literature already describes the separation of fatty acids in olein and super olein fractions on the basis of difference in MP (Khan et al. 2024). Concentrations of USFAs except EA in OP and SP were 94.07% and 0.78% while, MO had 43.9% USFAs. Low erucic mustard OP developed in this study contains the highest amount of USFAs than commercially significant sources of edible oils, sunflower and soybean has 85%–87% USFAs (Hamm et al. 2013). The degree of unsaturation in OP is also considerably higher than of sunflower and soybean oil reported in literature. To increase the amount of beneficial USFAs in the diet and for the development of functional foods, OP of MO can be used. Suitability of OP in the production of value‐added bakery products, margarine etc. its oxidative and thermal stability as a frying oil must be studied in detail. Out of 94.07% USFAs, 45.19% were C18:1, a monounsaturated fatty acid with proven oxidative stability in food systems (Aladedunye and Przybylski 2014). Ambient storage of MO, OP and SP revealed that content of USFAs significantly decreased 3 months (*p* < 0.05) while, saturated fatty acids remain unaffected (*p* > 0.05). Loss of C18:1, C18:2 and C18:3 in OP was 2.06%, 2.52% and 2.18%, respectively. Hussain et al. (2022) produced olein and super olein fractions from date seed oil, both had higher degree of unsaturation and experienced significant impact of storage phase on fatty acid profile.

**TABLE 2:** Fatty acid composition of MO, OP and SP.

Fatty acid	MO		OP		SP	
	0‐day	90‐days	0‐day	90‐days	0‐day	90‐days
C12:0	0.49 ± 0.03^B^	0.45 ± 0.06^B^	0.13 ± 0.04^C^	0.11 ± 0.02^C^	1.35 ± 0.16^A^	1.31 ± 0.06^A^
C14:0	2.63 ± 0.05^B^	2.55 ± 0.14^B^	0.09 ± 0.02^C^	0.08 ± 0.01^C^	4.38 ± 0.19^A^	4.32 ± 0.05^A^
C16:0	4.49 ± 0.16^B^	4.35 ± 0.18B	0.16 ± 0.03^C^	0.13 ± 0.03^C^	6.97 ± 0.37^A^	6.74 ± 0.38^A^
C18:0	3.27 ± 0.09^B^	3.16 ± 0.23^B^	1.29 ± 0.11^C^	1.19 ± 0.08^C^	5.67 ± 0.42^A^	5.51 ± 0.25^A^
C18:1	21.84 ± 0.24^C^	20.12 ± 0.41^D^	45.19 ± 0.28^A^	43.13 ± 0.46^B^	0.44 ± 0.26^E^	0.35 ± 0.29^F^
C18:2	13.95 ± 0.37^C^	12.42 ± 0.36^D^	30.76 ± 0.52^A^	28.24 ± 0.74^B^	0.11 ± 0.03D^E^	0.07 ± 0.01^F^
C18:3	8.11 ± 0.25^C^	7.31 ± 0.21^D^	18.12 ± 0.37^A^	15.94 ± 0.53^B^	0.23 ± 0.02^E^	0.15 ± 0.02^F^
C22:1	41.23 ± 0.56^C^	40.29 ± 0.15^D^	1.97 ± 0.09^A^	1.65 ± 0.07^B^	79.81 ± 0.98^E^	76.49 ± 2.64^F^

*Note:* Means of MO, OP and SP notified in a row with different letter, reveal statistically significant variation at 0.05 *p*‐value.

Abbreviations: MO, mustard oil; OP, olein; SP, stearin.

### 3.3 Phenolic Compounds

**FIGURE 1:**
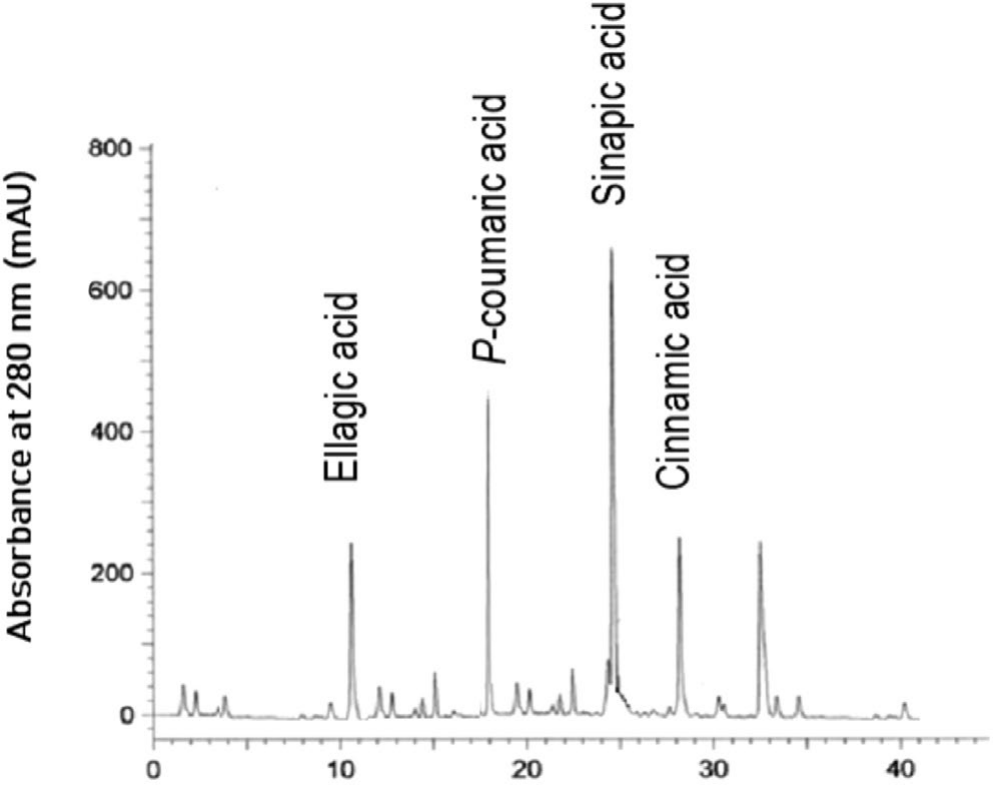
Phenolic compounds of olein portion of mustard oil.

**FIGURE 2:**
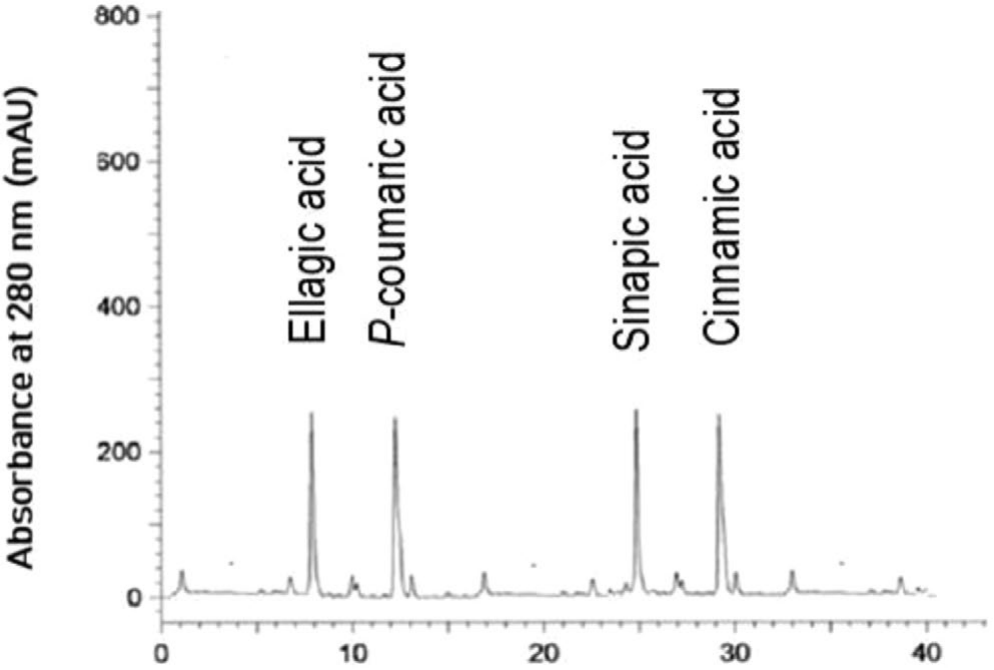
Phenolic compounds of stearin portion of mustard oil.

**FIGURE 3:**
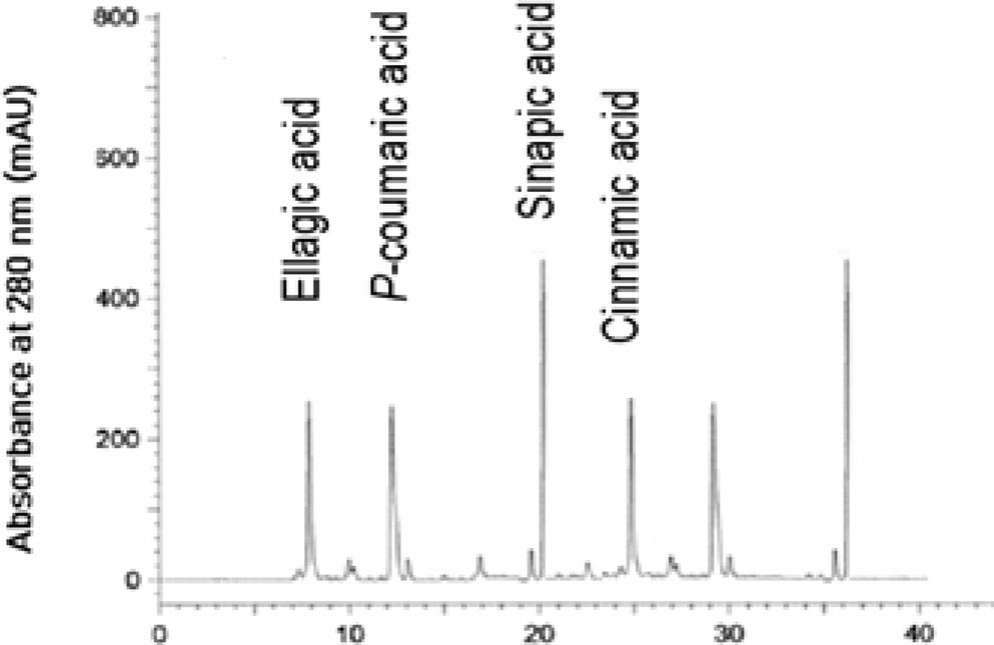
Phenolic compounds of mustard oil.

Based on the structure of the carbon chain, phenolic compounds can be classified into phenolic acids, flavonoids, and lignans in oils (Mikołajczak et al. 2021). As oil antioxidants, phenolic compounds have fetched a great deal of attention from oil chemists; a linear relationship between the stability of edible oils and total phenolic contents has been found (Franco et al. 2014). Further, phenolic compounds are responsible for the sensory and nutritional characteristics of lipids and can safeguard the oil from oxidative deterioration by quenching the free radicals in the auto‐oxidation phenomenon of lipid oxidation (Maqsood et al. 2014). HPLC analysis showed that sinapic acid, *p*‐coumaric acid, ellagic acid, and cinnamic acid were the major phenolic compounds in MO. Temperature‐assisted crystallization had a great impact on the distribution of phenolic compounds from MO to OP and SP. OP had the highest concentration of phenolic compounds, followed by MO and SP (*p* < 0.05). In OP, sinapic acid, *p*‐coumaric acid, ellagic acid, and cinnamic acid were 132.57, 236.19, 188.72, and 95.62 mg/100 g (Figure 1). In SP, sinapic acid, *p*‐coumaric acid, ellagic acid, and cinnamic acid were 45.13, 75.63, 39.87, and 15.24 mg/100 g (Figure 2). In MO, sinapic acid, *p*‐coumaric acid, ellagic acid, and cinnamic acid were 82.33, 174.31, 114.26, and 42.59 mg/100 g (Figure 3). The intensification/migration of higher concentrations of phenolic compounds towards the OP was due to the association of phenolic compounds with TAGs having low melting points. Low temperature crystallization of palm oil led to the migration of higher extents of phenolic compounds towards the fraction having a low melting point (Kumar and Krishna 2014). Strong statistical correlations were found between UM and phenolic compounds; fractions having higher UM content had higher phenolic content and vice versa (*R* ^2^ = 0.985 and 0.993). Phenolic compounds in MO were determined on HPLC; sinapic acid, *p*‐coumaric acid, ellagic acid, and cinnamic acid were the major phenolic compounds, and total phenolic contents of MO were 5.6 mg/100 g GAE/100 g. Total phenolic contents of MO analyzed in the present study are more than several edible oils (Nadeem and Imran 2016). The impact of winterization on phenolic compounds of chia oil was determined; most of the phenolic compounds were migrated towards the olein fraction, while stearin had a considerably lower number of phenolic compounds than olein fraction and parent chia oil (Ullah, Nadeem, Khalique, et al. 2016). Total phenolic contents of rapeseed oils ranged from 55.31 to 110.18 mg (FAE) (Symoniuk et al. 2018).

### 3.4 Phytosterols

Chemically, phytosterols belong to the group of organic compounds known as isoprenoids, these are the most significant nutraceuticals obtained from oilseeds and nuts, cereals also contain modest amount of phytosterols (Piironen et al. 2000). Structurally, phytosterols are analogues of cholesterol but functionally opposite, these are among the most significant nutritional constituent of dietary lipids. Phytosterols may decrease the intestinal absorption of dietary and biliary cholesterol by increasing its solubilization due to their capacity (Woyengo et al. 2009). Phytosterols are used in pharmaceutical sector for the production of steroids and in functional foods as an anti‐cholesterol food additive, intake of 1.5–3 g/day can reduce the LDL cholesterol from 8% to 15% to lower the risk of cardiovascular diseases, breast, pancreatic cancers, as an anti‐inflammatory, antiatherogencity activity (Fernandes and Cabral 2007). Food and Agriculture Organization has recommended that normal and hypercholesterolemic person should take enough (2–3 g/day) phytosterols to decrease cholesterol levels (Choudhary and Tran 2011). In oils, sterols are the major part of unsaponifiable matter, act as antioxidants in inhibiting the lipid oxidation, campesterol protect the oil from oxidation at elevated temperatures such as frying (Jin et al. 2018). Brassicasterol, ergosterol, campesterol, stigmasterol, β‐Sitosterol, Δ^5^‐Avenasterol, cycloartanol and cycloartenol contents in Chinese rapeseed oil were 2.54, 267.50, 2.83, 25.67, 394.11, 2.83, 40.92, 1.10 and 17.26 mg/100 g (Yang et al. 2019). Temperature‐assisted crystallization of MO significantly affected the distribution/migration of sterol in OP and SP (Table 3). GC–MS analysis showed that all the determined sterols moved/migrated towards OP. Highest concentrations of sterols were recorded in OP and lowest concentration of sterols were in SP. In temperature‐assisted crystallization of MO, migration of most of the sterols towards OP can be justified by the affiliation of sterols with TAGs having low melting points. Concentrations of brassicasterol in MO, OP and SP were 527.57, 652.33 and 124.19 mg/100 (*p* < 0.05). Concentrations of campesterol in MO, OP and SP were 2314, 2715 and 397.33 mg/100 g (*p* < 0.05). Concentrations of β‐Sitosterol in MO, OP and SP were 4387.37, 5469 and 1082.16 mg/100 g (*p* < 0.05). During the fractionation of palm oil, most of the phytosterols were migrated towards olein fraction (Tong et al. 2021). High oleic acid fraction of *Moringa oleifera* oil showed higher amounts of sterols than starting oil (Nadeem and Imran 2016).

**TABLE 3:** Phytosterols (mg/kg) of MO, OP and SP.

Sterol	MO	OP	SP
Brassicasterol	527.57 ± 1.42^B^	652.33 ± 1.19^A^	124.19 ± 0.97^C^
Campesterol	2314.10 ± 1.79^B^	2715.42 ± 1.55^A^	397.33 ± 1.14^C^
Campestanol	335.64 ± 0.88^B^	498.63 ± 2.34^A^	161.47 ± 1.23^C^
β‐Sitosterol	4387.37 ± 2.56^B^	5469.37 ± 3.47^A^	1082.16 ± 1.46^C^
Sitostanol	75.19 ± 0.61^B^	114.29 ± 0.69^A^	39.24 ± 0.46^C^
Δ^5^‐Avenasterol	756.97 ± 0.83^B^	956.10 ± 1.03^A^	198.82 ± 0.27^C^
Cycloartenol	115.62 ± 1.13^B^	165.93 ± 0.71^A^	48.79 ± 0.16^C^
24‐Methylene cycloartenol	69.14 ± 0.32^B^	116.77 ± 0.10^A^	47.13 ± 0.07^C^

*Note:* Means of phytosterols notified in a column with different letter, reveal statistically significant variation at 0.05 *p*‐value.

Abbreviations: MO, mustard oil; OP, olein; SP, stearin.

### 3.5 Antioxidant Capacity

Effect of temperature‐assisted crystallization on antioxidant capacity of OP and SP is documented in Table 4. Antioxidant capacity of OP and SP was dependent upon the distribution/migration of phenolic compounds and other antioxidative substances from MO to these fractions. Majority of the phenolic compounds were migrated from parent MO to OP therefore, OP had significantly higher values of TAC and DPPH. TAC and DPPH values were in the order of OP>MO>SP. In freshly produced OP, SP and MO, TAC values were 74.42%, 46.19% and 25.76% (*p* < 0.05). In freshly produced OP, SP and MO, DPPH values were 34.73%, 17.89% and 25.84% (*p* < 0.05). Ambient storage effect on TAC and DPPH values of OP, SP and MO were characterized for 3 months. In the storage phase, DPPH and TAC values remained non‐significant till 45 days. Testing TAC and DPPH values in OP, SP and MO at the end of storage indicated that TAC and DPPH values were significantly less than those recorded at 0 and 45 days. In 90 days old samples, TAC values of OP, SP and MO were 65.27%, 20.15% and 41.36% (*p* < 0.05). In 90 day old samples, DPPH values of OP, SP and MO were 27.13%, 10.12% and 18.34% (*p* < 0.05). Antioxidant capacity of food substrates is strongly correlated with oxidative stability, higher values of TAC and DPPH are usually linked with good storage stability (Sies 2007). Impact of temperature‐assisted crystallization on antioxidant capacity of olein fraction is documented, cottonseed was cooled, OP was separated from SP by filtration, OP showed significantly higher antioxidant capacity than SP (Azeem et al. 2015). OP of milk fat showed more antioxidant capacity than SP and substrate milk (Khan et al. 2019). Antioxidant capacity of pure safflower oil, first and second oleins were analyzed, antioxidant capacity successively increased from safflower oil to first and second oleins. Considerable increase in antioxidant capacity of first olein over safflower oil and second olein over first olein was due to the migration of phenolic compounds in first and second oleins (Khan et al. 2019). Storage impact (90 days) on OP and SP of date seed oil on antioxidant capacity was checked, antioxidant capacity of date seed oil its OP and SP significantly decreased in 3 months' storage (Hussain et al. 2022). Antioxidant capacity of rapeseed and olive oils were compared, olive oil showed higher magnitudes of phenolic compounds and strong antioxidant capacity than rapeseed oil (Szydłowska‐Czerniak et al. 2008). Antioxidant capacity of virgin and fractionated coconut oil was compared with each other, fraction showed more antioxidant capacity than original coconut oil (Marina et al. 2009). Oxidative stability of palm olein was superior to soybean oil due to the presence of a greater number of monounsaturated fatty acids than soybean oil (Memon et al. 2022). Carotenoids content of 15 examined samples ranged from 10.8 to 13.5 mg/kg, due to the antioxidant properties, carotenoids protect oils from lipid oxidation (Goulson and Warthesen 1999).

**TABLE 4:** Antioxidant capacity of MO, OP and SP.

Sample	Days	TAC (%)	DPPH (%)
MO	0	46.19 ± 0.09^C^	25.84 ± 1.19^C^
	45	45.77 ± 0.13^C^	24.91 ± 1.35^C^
	90	41.36 ± 0.24^D^	18.34 ± 1.44^D^
OP	0	74.42 ± 0.18^A^	34.73 ± 0.77^A^
	45	73.65 ± 0.37^A^	33.54 ± 0.91^A^
	90	65.27 ± 0.40^B^	27.13 ± 0.49^B^
SP	0	25.76 ± 0.72^E^	17.89 ± 0.32^E^
	45	24.63 ± 0.94^E^	16.33 ± 0.28^E^
	90	20.15 ± 1.06^F^	10.12 ± 0.36^F^

*Note:* Means of FFA and PV notified in a column with different letter, reveal statistically significant variation at 0.05 *p*‐value.

Abbreviations: MO, mustard oil; OP, olein; SP, stearin.

### 3.6 Lipid Oxidation

Chemically, oils and fats are triesters of glycerol (triglycerides), fatty acids when detached from glycerol molecule are known as FFA. Detachment of fatty acids may be catalyzed by lipases, moisture, temperature during storage and metal ions etc. Up to certain threshold level, FFA do not induce off‐flavor or bad smell in edible oils, as soon as FFA value reaches 0.2% oleic acid, a typical objectionable flavor is produced which is known as hydrolytic rancidity (O'Brien 2008). EU guidelines also suggest that FFA content of oils and fats should not exceed 0.2%. FFA can only be decreased in oil refineries by alkali or physical refining (Zhang et al. 2006). Edible oil processors should ensure the compliance of FFA as user industries cannot decrease FFA in their facilities. FFA content of OP, SP and MO in this investigation were 0.14%, 0.15% and 0.14% (*p* > 0.05) which is below than the permissible levels of EU (Table 5). Lower FFA in parent MO, its OP and SP were due to the extraction of fresh seeds (20 days old) with no heating/roasting. OP with lower levels of FFA and < 2% EA can be used as a cold pressed oil, it is highly advantageous for the food industries to use OP as an ingredient as refining, bleaching and deodorization is not required. Enormous amount of energy for the production of steam and operation of oil processing equipment is required to convert crude oils to processed oils. About 2 metric ton steam is required to produce refined, bleached and deodorized versions of vegetable oils (Fadda et al. 2022). All over the world, researchers are trying to develop virgin rapeseed oil, based on reasonable oxidative stability and < 2% EA, OP of MO, further research work would help the researchers to test its pharmacological effects in vivo and in vitro studies. OP had more than 95% unsaturated fatty acids out of which, 45.19 was oleic acid (C18:1). Scientifically, it is well established that monounsaturated fats have better oxidative stability than PUFA (Anwar et al. 2007). After the end of the storage phase of 3 months, FFA content of OP (> 95% unsaturated fatty acids) 0.17%, FFA content of SP (> 77% USFAs) at the same stage was 0.18% (*p* > 0.05). The non‐significant variation in FFA content of OP and SP during the long‐term storage of 3 months can be connected to the non‐correlation of FFA production factors with degree of unsaturation, factors affecting the FFA production is not based on the number of double‐triple bonds (Nor et al. 2008). During the long‐term storage of few vegetable oils, it was found that FFA content of MUFA and PUFA rich oils were not dependent upon degree of unsaturation (Fadda et al. 2022). Storage of 3 months significantly increased FFA; however, rise was induced by the age, reversible production pattern of FFA and several above‐mentioned factors with no correlation between FFA increase and degree of unsaturation. Transition in FFA content of olein fractions of date seed oil, palm oil, cottonseed oil and flaxseed oil during the storage phase has been well reported in literature. However, literature is silent on the production of FFA in OP of MO during the long‐term storage of 3 months. Stearin and olein fractionations were separated from dairy cream at 10°C, butter was produced from olein fraction and stored for 3 months, FFA increased from 0.08% to 0.14% in 3 months storage (Nadeem et al. 2014). Frega et al. (1999) discovered a statistically strong correlation between FFA and auto‐oxidation of lipids. In present investigation, OP of MO were developed to provide a new source of edible oil that possesses its traditional flavor and lower EA content. FFA of rapeseed oil samples ranged from 0.3% to 1.2%, Bureau of Indian Standard for edible vegetable oil allow FFA up to 5% (Johnson et al. 2009). Food industries prefer to use those versions of vegetable oils that can with stand longer phase of storage without undergoing lipid oxidation. PV analysis was included in this research work due to its worldwide acceptability, accuracy as an official method of the American Oil Chemists Society. From peroxide value, the status of lipid oxidation of oils and fats can be adjudged and future storage stability may be anticipated (Musakhanian et al. 2022). Regulatory guidelines of EU suggest that PV of oils, fats and foods should not be more than 10 (MeqO_2_/Kg). Conversion of MO to OP and SP showed a non‐significant trend on PV (Table 5). In fresh condition, PV of MO, OP and SP was 0.65, 0.68 and 0.71 (MeqO_2_/Kg). PV after 3 months of ambient storage of MO, OP and SP was 2.56, 3.84 and 1.35 (MeqO_2_/Kg). For industrial usages, results of PV of OP is highly encouraging as it is within the permissible levels of EU. Reasonable storage stability of OP till 3 months of storage (> 95% UFAs) can be connected to presence of higher amounts of phenolic compounds and natural antioxidants. PV of soybean, sunflower and canola oils increased during the storage phase (Zaunschirm et al. 2018). Nadeem, Imran, Taj, et al. (2017) found that PV of mango kernel oil increased in 3 months ambient and accelerated storage. After 90 days of storage at 25°C, PV of cold extracted MKO samples ranged from 0.25 to 7.35 (mg GAE/g). Oxidative stability of OP, SP and MO was determined on Rancimat with the assistance of Stab Net software. Induction period of OP, SP and MO were 6.49, 3.62 and 12.39 h, respectively (at 120°C). Induction period of rapeseed oil samples ranged from 13.10 to 14.12 h (Szterk et al. 2010). The lower oxidative stability of OP and MO in current study was due to the presence of about 98% USFAs and measurement of induction period at 120°C. However, induction period of SP was comparable to the values reported in literature. Induction period of rapeseed oil was more than flaxseed oil (Madawala et al. 2012). Oxidative stability of lipids is based upon composition of fatty acids; higher degree of unsaturation leads to the lower oxidative stability (Zhang et al. 2006). Khan et al. (2019) determined the induction period of olein and unmodified fat, olein fraction greatly showed lower induction period due to the intensification of unsaturated fatty acids. Olein fraction had a greater number of PUFA thus showed inferior oxidative stability (Nadeem et al. 2015).

**TABLE 5:** Lipid oxidation of MO, OP and SP.

Sample	Days	FFA%	PV (MeqO_2_/kg)	Induction period (hours)
MO	0	0.14 ± 0.02^C^	0.65 ± 0.05^F^	6.49 ± 0.04^C^
	45	0.15 ± 0.01^B^	1.15 ± 0.12^D^	5.91 ± 0.13^D^
	90	0.16 ± 0.04^A^	2.56 ± 0.19^B^	4.52 ± 0.08^F^
OP	0	0.14 ± 0.02^C^	0.68 ± 0.16^F^	3.62 ± 0.15^G^
	45	0.15 ± 0.03^B^	1.47 ± 0.24^B^	3.28 ± 0.06^H^
	90	0.17 ± 0.01^A^	3.84 ± 0.27^A^	2.72 ± 0.03^I^
SP	0	0.14 ± 0.04^C^	0.71 ± 0.02^F^	12.39 ± 0.21^A^
	45	0.44 ± 0.02^B^	0.95 ± 0.04^E^	11.80 ± 0.25^B^
	90	0.18 ± 0.05^A^	1.35 ± 0.07^C^	8.61 ± 0.32^E^

*Note:* Means of FFA and PV notified in a column with different letter, reveal statistically significant variation at 0.05 *p*‐value.

Abbreviations: MO, mustard oil; OP, olein; SP, stearin.

### 3.7 Sensory Evaluation

**FIGURE 4:**
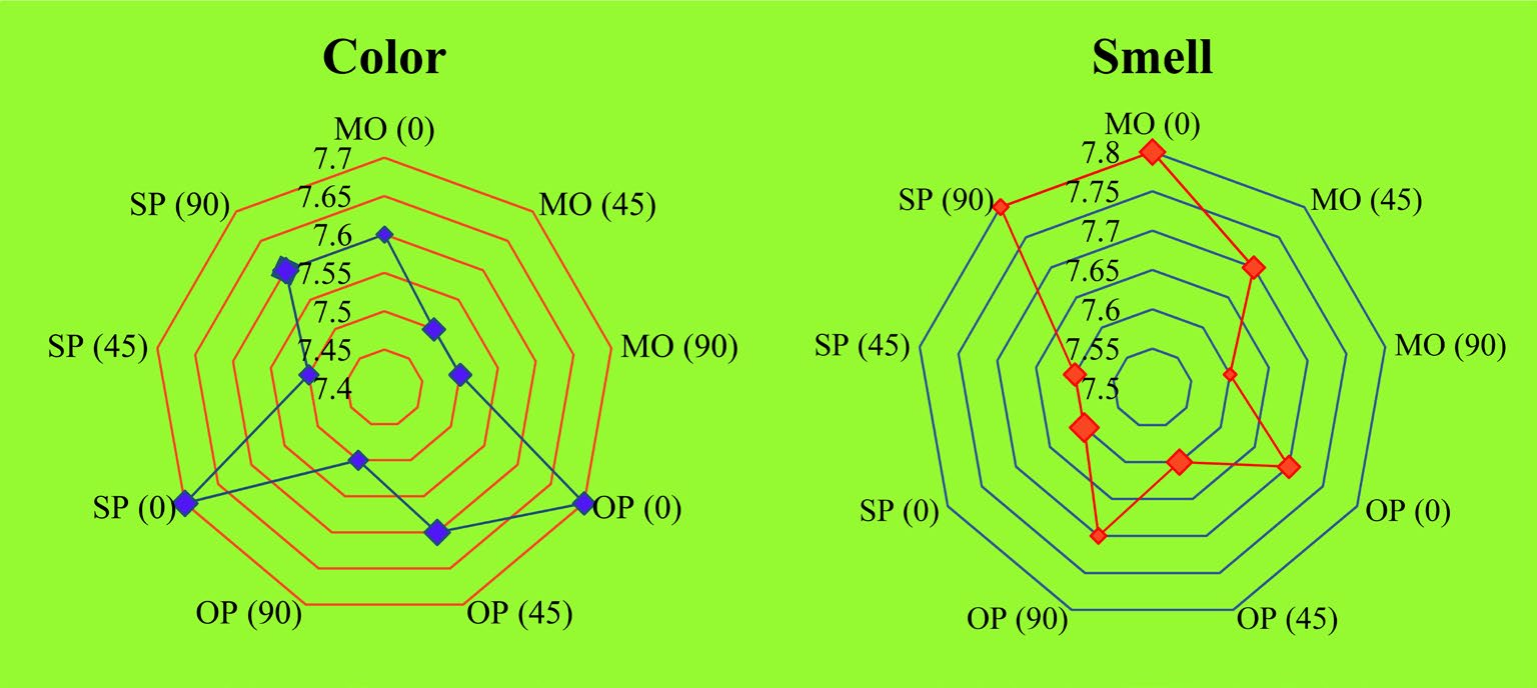
Sensory evaluation at 0, 45 and 90 days.

In a specialized sensory evaluation laboratory, no difference in color and smell of MO, OP and SP was found when tested at the stages of 0, 45 and 90 days with no objection of having oxidized flavor (Figure 4). Color scores of MO, OP and SP was 7.6, 7.7 and 7.7 and smell scores MO, OP and SP was 7.8, 7.7 and 7.6, respectively. Lower color of OP and SP may be useful for food applications. OP and SP were smelling like typical OM. Safflower oil was subjected to dry crystallization to fractionate in olein and stearin, color and smell scores of both the fractions were similar to the safflower oil (Khan et al. 2024). Medium, low and very low melting point fractions of milk fat were produced by cooling to 25°C, 15°C and 10°C temperatures, several variations in composition, fatty acid profile and sterols were found however, sensory properties of all the fractions were non‐significantly different from the milk fat (Khan et al. 2019).

## 4 Conclusion

EA content of MO was decreased to < 2% level using temperature‐assisted crystallization with no effect on FFA, peroxide value, and color of MO and its olein and stearin variant. Olein, which was rich in phenolic compounds, had a higher antioxidant capacity than MO and stearin. After the completion of the storage phase, FFA and PV of OP (low EA variant) were within the limits of European Union. Temperature‐assisted crystallization can be used to produce a low EA variant.

## Author Contributions

**Eliasse Zongo:** writing – review and editing, funding acquisition, visualization. **Muhammad Nadeem:** conceptualization, methodology, validation, formal analysis, investigation, resources, writing – original draft, supervision. **Mohamed Fawzy Ramadan:** writing – review and editing, validation. **Layla A. Alahmari:** funding acquisition, writing – review and editing. **Muhammad Abdul Rahim:** conceptualization, methodology, writing – original draft, writing – review and editing, visualization, project administration.

## Funding

The authors extend their appreciation to the Deanship of Scientific Research at Northern Border University, Arar, KSA for funding this research work through the project number NBU‐FFR‐2026‐2273‐02.

## Ethics Statement

The authors have nothing to report.

## Consent

All authors have given their full consent to participate.

## Conflicts of Interest

The authors declare no conflicts of interest.

## Data Availability

The data that support the findings of this study are available from the corresponding author upon reasonable request.
